# Social Agency as a continuum

**DOI:** 10.3758/s13423-020-01845-1

**Published:** 2020-12-07

**Authors:** Crystal A. Silver, Benjamin W. Tatler, Ramakrishna Chakravarthi, Bert Timmermans

**Affiliations:** School of Psychology, School of Psychology, William Guild Building, Kings College, University of Aberdeen, Old Aberdeen, AB24 3FX UK

**Keywords:** Sense of Agency, Social Agency, Joint action, Social interaction, Cooperation

## Abstract

Sense of Agency, the phenomenology associated with causing one’s own actions and corresponding effects, is a cornerstone of human experience. Social Agency can be defined as the Sense of Agency experienced in any situation in which the effects of our actions are related to a conspecific. This can be implemented as the other’s reactions being caused by our action, joint action modulating our Sense of Agency, or the other’s mere social presence influencing our Sense of Agency. It is currently an open question how such Social Agency can be conceptualized and how it relates to its nonsocial variant. This is because, compared with nonsocial Sense of Agency, the concept of Social Agency has remained oversimplified and underresearched, with disparate empirical paradigms yielding divergent results. Reviewing the empirical evidence and the commonalities and differences between different instantiations of Social Agency, we propose that Social Agency can be conceptualized as a continuum, in which the degree of cooperation is the key dimension that determines our Sense of Agency, and how it relates to nonsocial Sense of Agency. Taking this perspective, we review how the different factors that typically influence Sense of Agency affect Social Agency, and in the process highlight outstanding empirical questions within the field. Finally, concepts from wider research areas are discussed in relation to the ecological validity of Social Agency paradigms, and we provide recommendations for future methodology.

Sense of Agency is the phenomenology associated with the responsibility we feel over voluntary actions and their effects: You click a hyperlink on a web page, and the new page opens. If this takes too long, you will feel frustration, and once the page opens, you may feel less responsible for it happening. If a pop-up advertisement window opens, you may feel little Sense of Agency, or on the contrary be acutely aware that it was you who clicked a dodgy link. Social Agency is Sense of Agency when the voluntary action’s effect is the direct or indirect reaction of a conspecific that we perceive as an independent agent. Walking along a street, you smile towards a friend you see, which causes them to return your smile in kind. Consider also if instead of smiling at the oncoming person, you smile at the dog walking beside them. This may still indirectly make the other person smile, even though they are not the target of your action.

Social Agency has vital importance to social development. Humans are an intrinsically social species, and the majority of our actions elicit social effects (Pfeiffer et al., 2014) alongside environmental effects, irrespective of intention. From infancy, humans are attuned to detect (Hains & Muir, 1996) and respond to social cues (Michel, Wronski, Pauen, Daum, & Hoehl, 2019), and we prefer prosocial responses from others (Grynszpan, Martin, & Fossati, 2017). We also ubiquitously engage in actions where social effects are the *exclusive* objective (Gobel, Tufft, & Richardson, 2018). This is key during development, where social interactions transmit information initially through dyadic interaction, and then through triadic interaction with another agent towards a third object or person (Sebanz, Bekkering, & Knoblich, 2006). This social transmission of information, or ‘social learning’ (Whiten, 2017), contributes to the development of the Self. Social learning in humans also has adaptive value by facilitating exponential cultural growth, and introduces a degree of interdependence not found in any other species (Mesoudi & Thornton, 2018).

Despite social interaction being crucial to the development of social understanding (Schilbach et al., 2013; Whiten, 2017), the empirical investigation of Social Agency is still very much in its infancy, and its neurobiological exploration almost nonexistent (but see Buchholz, David, Sengelmann, & Engel, 2019; Dumas, Martinerie, Soussignan, & Nadel, 2012). In the extant literature, the term “Social Agency” is loosely used to designate the Sense of Agency in quite distinct situations, such as human–computer interactions, joint action situations, or more diffuse social interactions, without necessarily distinguishing how these situations differ. Here, we bring together these different instantiations of Social Agency and, based on the stark empirical differences in the nature of Social Agency in such situations, propose a novel approach that integrates social and nonsocial agency in one framework. This framework will be based on the underlying properties of the context in which the consequences of the action occurs, on the characteristics of these consequences, and on the beliefs held about the other agent by the person executing the action.

This review first introduces Sense of Agency, and more specifically, Social Agency as a core element of the human experience. Following, we develop a comprehensive picture of the concept of Social Agency. Currently, the empirical view of Social Agency is oversimplified as a uniform construct that applies to all forms of social situations and as such, conflicting results are abundant in the literature. Using an evidence-based approach, this review will instead argue that Social Agency must be viewed as a continuum, where differing contextual and experimental elements influence agency. Specifically, we propose that the degree of cooperation between actors within a social interaction is a key dimension which influences Sense of Agency: The presence of cooperative elements within an interaction enhances agency, whereas social interaction with little to no cooperation diminishes agency.

Re-representing Social Agency as a continuum reconciles existing empirical disparities and crucially highlights unexplored areas within Social Agency as well as unanswered empirical questions brought up by the existing literature. Addressing these gaps and discrepancies would enrich our understanding of Social Agency. A fallout of our model is that it suggests clear experimental approaches to resolve these issues. The review will summarize the outstanding empirical questions for Social Agency and give recommendations for future research.

## Sense of Agency and Social Agency

Sense of Agency is the experience of responsibility over voluntary actions and their effects (Haggard, 2017; Haggard & Chambon, 2012). A typical example is the responsibility felt over a bulb lighting up when a switch is pressed, or over a ball moving when it is kicked. Sense of Agency is the construct by which we phenomenologically distinguish self-induced actions from actions due to other causes (see Hoerl et al., 2020). If both you and another reach to press the light switch at the same time, what processes are used to determine who caused the bulb to illuminate? Sense of Agency relies on either action prediction or action–outcome comparison (Haggard & Chambon, 2012). Action prediction induces Sense of Agency when a predicted outcome matches the experienced sensory feedback (Frith, 2012). Action–outcome comparison is postdictive, inducing Sense of Agency when the experienced outcome, when compared with the action, allows an inference of causality (Moore & Obhi, 2012).

Sense of Agency is how we identify our own actions in the world (Hoerl et al., 2020), and is argued to be a cornerstone of human experience (Haggard, 2017; van Hateren, 2015). Abnormalities in agency have been identified as a contributing factor in an increasing number of clinical disorders (Schimansky, David, Rössler, & Haker, 2010; Timmermans & Schilbach, 2014). Depression is associated with deficient Sense of Agency (Haggard & Chambon, 2012), whereas schizophrenia is associated with both deficient and misattributed agency (Garbarini et al., 2016). These disparities are argued to induce feelings of helplessness or loss of volition (Haggard & Chambon, 2012). Agency is also of cardinal importance among nonclinical populations because of its central role in society as the foundation for legal responsibility; societies take Sense of Agency into account when holding individuals responsible for unlawful acts (Haggard, 2017; Hallett, 2018; Tsimploulis, Niveau, Eytan, Giannakopoulos, & Sentissi, 2018).

Crucially, whereas action outcomes and their anticipation are often closely linked to our naïve theories of physics and other material interactions (e.g. electricity), this is not necessarily always the case. Your actions may also cause a corresponding action from someone else, for example having another follow your gaze to an intended object or laugh at your joke. This Social Agency, the responsibility felt over a social effect, can be construed as a specific kind of Sense of Agency (Brandi, Kaifel, Bolis, & Schilbach, 2019), with important distinctions that up until now have remained underspecified. One such distinction between social and nonsocial contexts is that social interactions are much less predictable. Social effects are produced by an independent agent acting under their own volition and therefore contain inherent variance, reducing expectations in relation to action–effect fluency (Pfeiffer et al., 2014; Stephenson, Edwards, Howard, & Bayliss, 2018). We would expect a light to illuminate immediately upon pressing the switch, whereas when leading another’s gaze, we expect them to respond at a time and in a way of their choosing; in fact, when another person reacts too quickly to our actions, we may feel less as the cause of their actions (Pfeiffer et al., 2012). This is relevant to Social Agency specifically as the predictability of the social actor you are interacting with can modulate self-Social Agency (Bolt & Loehr, 2017).

This element of high variance in social interactions illustrates why Social Agency should not simply be viewed as a uniform construct. The temporal variance is unpredictable and is modulated by fluid social influences, and can therefore influence Sense of Agency in different ways. Social Agency will hence be experienced distinctly in different social contexts. Most importantly, this is only one example of many complex components inherent in social interactions, which may all influence Sense of Agency. This review will identify key dimensions of social interaction that influence agency and propose that Social Agency forms a continuum centred on one principal dimension: cooperation.

### What is Social Agency?

Explaining Social Agency as the responsibility over a social effect, as in the previous section, is in itself an oversimplification of the construct, which in reality encompasses any situation in which any social aspects have a bearing on how much Sense of Agency you will experience. Another person laughing at your joke provides an easily understood example of what constitutes a social effect. In those circumstances, Social Agency is very much individual: Your action causes an effect produced by another (Social Agency [effect]), even if in reality pure isolationist examples of action–reaction are probably rare. What this example neglects to identify, however, is that there are other distinct circumstances in which Social Agency can arise. A second instance of Social Agency is when two or more individuals act together, forming a joint identity (Social Agency [joint]). For example, when two people reposition a sofa within a room, it can be construed that *they* are moving the sofa. It is this joint identity which is the defining factor in creating Social Agency under these circumstances, with the effect, the goal of the joint action, not necessarily being social in nature. Finally, Social Agency can simply arise from acting in a social context: being in the presence of another independent agent (Social Agency [context]). Consider, for example, completing the crossword of a newspaper with someone glancing over your shoulder at your progress. In this instance, the other is neither the target of the action (as in [effect]), nor involved in the action (as in [joint]), but their mere presence could influence your Sense of Agency.

It is important to note that “effect,” “joint,” and “context” should not be seen as categorically distinct types of Social Agency, but rather as different dimensions along which a given situation can vary, and which are neither dependent on each other, nor mutually exclusive. For instance, while moving a sofa, even if the intended primary outcome is distinctly nonsocial, there may be secondary outcomes (stronger feeling of affiliation) that are social, and furthermore the way I am moving with the sofa will, mediated by the overarching action goal, influence the other’s movements, also constituting a social effect, just as my actions are their effect. It can even be argued that the mere copresence of two agents, even if they do not directly or indirectly influence each other’s behaviour, changes their action affordances in that space (Gibson, 2014), without the need for specific joint actions or social action effects. Drawing such distinctions lies outside of the scope of this review. Instead, the three proposed dimensions will be used to distinguish different ways in which research has operationalized Social Agency, leading to divergent results.

Before delving into the details, we would like to note that, like its nonsocial equivalent, Social Agency is also a layered construct, albeit with more nuances due to its social nature. Low-level, sensorimotor processes inform our prereflective *feeling* of agency (Balconi, 2010; Metcalfe & Greene, 2007). This is debatably correlated (Imaizumi & Tanno, 2019) to, only weakly correlated (Moore, Middleton, Haggard, & Fletcher, 2012) to, or even dissociated (Dewey & Knoblich, 2014; Grynszpan et al., 2019; Obhi & Hall, 2011a; Saito, Takahata, Murai, & Takahashi, 2015) from our *judgement* of agency, which is a higher-level and reflective process operating through belief-like propositions (Balconi, 2010). An additional, uniquely social, aspect is an *evaluative* component to this higher level of Sense of Agency, where a sense of moral responsibility causes the evaluation of actions against sociocultural norms (Balconi, 2010; Montague & Lohrenz, 2007). For example, when playing music on public transport, the normative behaviour is to use earphones as to not disturb others, whereas listening alone does not require that evaluation. This evaluative component has recently been found to be dissociable from *feeling* agency (Caspar, Lo Bue, Magalhães De Saldanha da Gama, Haggard, & Cleeremans, 2020), and can reduce self-responsibility when coerced to perform reprehensible actions. For example, when following an order to administer pain to another, less agency is experienced than when freely choosing to perform the same act (Caspar, Cleeremans, & Haggard, 2018).

While we would like to stress the importance of different levels of agency experience, this review will focus on *feeling* and *judgement* of agency, as these are most widely present in the existing body of research, even if sometimes used interchangeably. For both of these levels, the review will address, in a unified manner, studies which scrutinize all three instances of Social Agency (“effect,” “joint,” and “context”). What will become apparent as the review builds, is that specifically Social Agency (effect), which is arguably a core aspect, in that it may underly all three dimensions, is in and of itself critically under researched.

### Issues with defining and measuring Social Agency

#### Conflicting views on Social Agency

As noted above, the different instances of Social Agency are neither dependent on each other nor are they mutually exclusive. Hence, it will come as little surprise that there are widespread disparities in the research and findings on Social Agency, and particularly regarding the definitions of the overall construct. These disparties also manifest as a lack of theories on how specific types of Social Agency relate to each other. Here, we will use a few representative examples to flesh out these concerns:

- Beyer, Sidarus, Bonicalzi, and Haggard (2017) state that “the presence of others agents can lead to reduced outcome monitoring and a reduction in individual sense of agency, even in the absense of attributional ambiguity.” (p. 144). This stance seems to predict universal reduced Social Agency effects with or without attributional ambiguity, which is not the case (see Obhi & Hall, 2011a).
- Pfister, , Obhi, Rieger, and Wenke (2014) limit inferences to only *predictable* social effects, stating that Sense of Agency “not only occur[s] for physical effects in the environment, but also for social action effects, i.e. predictable actions of other agents” (p. 9).
- Authors investigating joint action or cooperative interaction also generally restrict their inferences and describe Social Agency effects within a narrow framework. In joint action, Obhi and Hall (2011a) infer that “when two individuals are involved in bringing about some effect, a ‘we’ identity is automatically (i.e. pre-reflectively) formed, and both register agency” (p. 662). This does not situate this specific instance of Social Agency within the wider field, and hence a cohesive and comprehensive picture of the construct cannot be developed.
- Finally, in a recent theoretical review, Brandi et al. (2019) offer a surprisingly broad, and hence vague, definition of Social Agency: “the sense of self that is gained through the perceived control one exerts over the social world” (p. 18). Previous work rebuts this definition, where it can be the intrinsic *lack* of control (i.e. the unpredictability of social outcomes) which induces Social Agency effects (Beyer et al., 2017; Beyer, Sidarus, Fleming, & Haggard, 2018).

It is this lack of cohesion which the proposed continuum framework seeks to address.

#### Difficulties in measuring Social Agency

Social Agency is not only difficult to define, but also to measure. There are intrinsic characteristics of social interactions which confound traditional (nonsocial) measures. For example, measuring Sense of Agency explicitly (i.e. *judging* agency) through self-report in joint action is challenging. Direct, explicit measures rely on reporting the self-attribution of action effects: the extent to which one judges oneself as an agent of a particular action and its consequence (Moore et al., 2012), but when individuals act together, this judgement is not clear cut, which might reflect the inherent variance and uncertainty in judging Social Agency. Explicit measures are also susceptible to cognitive self-biases, such as the overestimation of self-agency (Tsakiris, Haggard, Franck, Mainy, & Sirigu, 2005; Wegner & Wheatley, 1999). These factors make it difficult to unambiguously measure self-agency or joint-agency effects in joint action (for discussion of methodoligcal issues, see Bolt, Poncelet, Schultz, & Loehr, 2016; Dewey & Knoblich, 2014; Le Bars, Devaux, Nevidal, Chambon, & Pacherie, 2020).

The most commonly used implicit measure (i.e. *feeling* agency: inferring agency from perceptual effects; Imaizumi & Tanno, 2019) in Social Agency paradigms is Temporal Binding: the subjective compression of the perceived time interval between an action and its effect (Beck, Di Costa, & Haggard, 2017; Buehner, 2012; Engbert, Wohlschläger, Thomas, & Haggard, 2007). Temporal Binding is traditionally measured in two ways: estimation of the perceived interval between action and effect (Engbert et al., 2007) or judgements of the perceived time of either action or effect separately (Libet, 2002). Only the interval estimation method, where participants verbally (Engbert et al., 2007), or through motor replication (e.g. press and hold the space bar on a keyboard; Stephenson et al., 2018), estimate the interval between action and effect within the same trial, is possible in Social Agency paradigms.

Whilst interval estimation gives a general measure of time compression, Temporal Binding is argued to involve separate *action shifts*, where perceived action is later than actual action and/or *outcome shifts*, where perceived effect is earlier than actual effect. Therefore, using separate action and effect judgements allows for inspection of these components, which possibly have different underlying mechanisms (see Tanaka, Matsumoto, Hayashi, Takagi, & Kawabata, 2019), and is arguably the superior measure (for review, see Wen, 2019). Unfortunately, the standard time-point judgement method cannot be easily used in Social Agency paradigms, as the need to constantly monitor visual informaiton (i.e. an on-screen clock face with rotating hand; Libet, 2002) greatly restricts participants from focusing on any other visual, and indeed nonvisual, information, such as the actions of an interaction partner. Hence, in its current formulation, this method appears unsuitable to assess the temporal binding elicited by Social Agency. However, due to the greater specificity afforded by the time-point judgement method, it would be prudent for future Social Agency studies to determine whether an alternative method of assessing time of events, such as using an auditory oscillation or metronome instead of a visual clockface, might be more amenable for measuring temporal binding.

These complications may help to explain the somewhat limited measurement methods for Social Agency, compared with the wider set of options available to examine nonsocial Sense of Agency. What is yet to be addressed, however, is how specific experimental factors affect Social Agency, which will be comprehensively covered in the section titled Factors Influencing Sense of Agency.

## Types of Social Agency

As reiterated throughout this review, it is crucial that an evidence-based approach is used when building a comprehensive model of Social Agency. Instead of trying to fit diverging results to the same construct, resulting in widespread disagreements in the field, it is prudent to categorize the existing literature by agency effects (enhancing or diminishing) to extract key elements or dimensions which these paradigms share. The strongest enhancement of agency is found in paradigms where there is a high degree of cooperation between actors, inducing a joint Social Agency (i.e. Joint Agency). Joint Agency is subdivided into instances of ‘*we*’ agency (Obhi & Hall, 2011a), where the blurring of self–other distinction enhances agency past the boundaries of the Self, and ‘*shared’* agency, where self–other distinction remains intact, creating a dual sense of agency where one’s self agency and Joint Agency coexist (Pacherie, 2012). Determining which type of Joint Agency manifests in a paradigm relies, crucially, on whether there is ambiguity over whose action causes an effect. There are further paradigms where agency effects are dependent on the agent’s role in a cooperative interaction. In cooperative tasks with clearly defined hierarchical roles, vicarious agency can be induced for the dominant actor over the subordinate’s actions. Conversely, in such instances, the subordinate actor’s agency is violated, resulting in diminished agency. Finally, ‘*interfered’* agency is found in paradigms where there are ill-defined goals or little or no cooperation between actors. In these instances, it is the monitoring of predicted or actual actions of the other which diminishes agency by interfering with self-related processes (Beyer et al., 2017; Beyer et al., 2018).

Each of these categories will be explored in this section. The data and observations from studies implementing these distinct paradigms provide the foundation for the development of the concept of Social Agency as a continuum, centred on the key dimension of cooperation. This concept will be explained in detail. The concept will also encompass traditional nonsocial agency so that comparison may be made between that and the Social Agency effects. As natural social interactions are endless in variation, limitations of the continuum framework will then be outlined in relation to the types of interactions currently being studied (e.g. structured, transient).

### ‘We’ agency

We are often in circumstances where we must cooperate with others to achieve shared goals (joint action goal; Clarke et al., 2019; Sebanz et al., 2006). This may be at an individual (i.e. with interaction partners) or group level. Importantly, in joint action contexts, cooperative physical actions do not have to be congruent to facilitate performance: Sebanz et al. (2006), state that joint action is “any form of social interaction whereby two or more individuals coordinate their actions in space and time to bring about a change in the environment” (p. 70). This is true as long as incongruent actions between interaction partners are complementary to the shared aim (Clarke et al., 2019; Sartori & Betti, 2015). Interestingly, later in the same paper, when describing agency in the context of joint action, Sebanz et al. (2006) cast agency as a problem of causal ambiguity: “In joint action, such problems of agency arise when one’s own and others’ actions are carried out at approximately the same time and result in similar effects” (p. 75). In doing so, they clearly indicate that this interaction where action origin is ambiguous is a specific form of joint action, whereby two cooperating individuals do not just share an overarching joint action goal, but do so to a degree where their individual actions have a joint and near simultaneous effect on the environment.

The action ambiguity Sebanz et al. (2006) speak of is one factor which can blur self–other boundaries to the point of inducing ‘we’ agency. In a joint-action task which introduced an element of ambiguity, it was found that both initiator and responders exhibit Temporal Binding effects over their own actions, but only the initiator reported explicit agency (Obhi & Hall, 2011a). Participants were asked to perform a key press at a time of their choosing 1–6 seconds after onset, and after a further 200 ms, a tone would sound. Once the initiator performed the key press, the responder was required to immediately make an equivalent key press. Measures of Temporal Binding and feeling of causality over the effect (i.e. explicit agency) were taken on each trial. Temporal Binding, but not explicit agency, was observed in both participants both when the roles were assigned on a block-wise basis *and* also when they were established dynamically trial-wise by who acted first. The dynamic condition greatly increased the ambiguity over whose action caused the effect, inducing ‘we’, rather than shared agency. The authors, indeed, argued that their findings arose from the automatic formation of a joint agentic identity with blurred self-ness (Obhi & Hall, 2011a).

Moving unintentionally in time with another (i.e. interpersonal rhythmic coordination or ‘social synchrony’; Kinreich, Djalovski, Kraus, Louzoun, & Feldman, 2017; Launay, Tarr, & Dunbar, 2016; Reddan, Young, Falkner, López-Solà, & Wager, 2020; Tarr, Launay, & Dunbar, 2016) can also produce the action origin ambiguity which induces ‘we’ agency to form between parties. In a task where participants moved their arm in time with a metronome, a confederate, visible on-screen, moved either synchronously or asynchronously at the same time (Reddish, Tong, Jong, & Whitehouse, 2020). Participants believed the confederate to be a second participant who was either listening to the same or different metronome rhythm. The ostensible purpose of the study was to understand how people interact over video. When moving synchronously, participants extended their own agency to that of the other’s actions, experiencing a degree of agency over these. Taken alone, this could be attributed to vicarious agency—that is, the misattribution of another’s action to the self (see the section titled Vicarious and Violated Agency), but interestingly, synchrony was also found to lead participants to report the other having a degree of agency over their own actions. This indicates that some form of Joint Agency (arguably, ‘we’ agency) was formed. Reddish and colleagues (2020) concede that this was contrary to the prediction derived from empirical findings of synchrony with a nonagentic entity, which again establishes Social Agency as distinct from Sense of Agency more generally (see Kalckert & Ehrsson, 2012, 2014).

Interestingly, it appears that ‘we’ agency is not established when performing joint action with a robotic partner (Grynszpan et al., 2019). This is part of the wider discussion into whether Social Agency effects are human centric and so will be elaborated on in the section titled Social Agency With Artificial Agents.

### Shared agency

In interactions where the self–other distinction is intact, but agents engage in joint action, shared agency is induced. Initiating joint attention with another by leading their gaze is a socially crucial instance of this, which has been studied extensively using eye-tracking methodology (for review, see Caruana, McArthur, Woolgar, & Brock, 2017b).

Leading the gaze of an on-screen face to an intended object was demonstrated to increase Temporal Binding compared with a control condition with a passive face (Stephenson et al., 2018). Explicit measures mirrored implicit findings, with increased agency ratings for active, compared with passive, faces (Stephenson et al., 2018). Interestingly, Temporal Binding was also increased for a condition where joint attention was achieved without gaze leading. Participants looked towards the intended object at its onset and the on-screen face followed their gaze *incidentally*. This would imply that even initiating joint attention without intention can enhance implicit agency (Stephenson et al., 2018). This would suggest that *how* shared agency is established is not important to the phenomenology of the initiator. However, it is not clear whether the same Sense of Agency would be experienced if the participant’s gaze was led towards the object. Hence, the generalizability of the phenomenology of joint attention for all parties remains untested. Participants in this study also never believed the face on-screen was anything other than computer generated, limiting how social the interaction was perceived. The agency behind computer-generated anthropomorphic characters is an important design consideration when scrutinizing social phenomenon and shall be discussed in the section titled Social Agency With Artificial Agents.

As mentioned previously, some level of coordination is present in all joint action. In a joint-action task, a coordination manipulation demonstrated that instances of mutual coordination between actors increased the level of shared control over the task, compared with instances where only one of the actors had to coordinate with the other (i.e. asymetrical coordination; Bolt et al., 2016). This demonstrates that it is the *mutuality* of coordination, which includes action monitoring and perceptual predictability of the other’s actions and its consequences, that induces a sense of shared agency (Bolt et al., 2016; Pacherie, 2014).

### Vicarious and violated agency

Joint-action tasks vary in how egalitarian or hierarchical they are (Pacherie, 2012), which can impact Social Agency. Within the literature, there is evidence supporting vicarious agency (Clarke et al., 2019; Wegner & Wheatley, 1999; Wegner, Sparrow, & Winerman, 2004), and enhanced self-agency (Obhi & Hall, 2011a; Reddish et al., 2020; Weiss, Herwig, & Schütz-Bosbach, 2011) in egalitarian joint-action tasks. Vicarious agency is when authorship of an action performed by another is misattributed to the self (Wegner & Wheatley, 1999; Wegner et al., 2004). It has also been found that during egalitarian joint action, implicit Sense of Agency (e.g. Sensory Attenuation: reduction in the perceived phenomenological experience of external events; Beck et al., 2017; Weiss et al., 2011) increases for both self-action and other-action effects (Weiss et al., 2011).

**Fig. 1:**
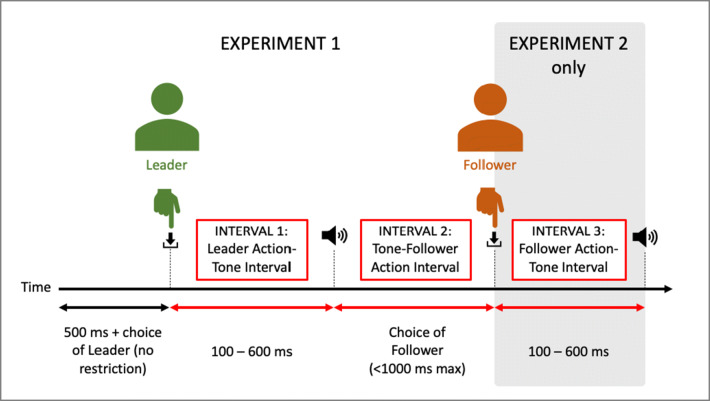
Modified representation of the Pfister et al. (2014) trial sequence for Experiments 1 and 2 (grey section Experiment 2 only), showing timing information, actions according to actor roles, and tones

Vicarious agency cannot be solely attributed to circumstances of Joint Agency. Vicarious agency is also exhibited in hierarchical interactions, for dominant actors (Pfister et al., 2014). Self and other Temporal Binding effects were scrutinized in two experiments with well-defined hierarchical roles, where participant pairs were assigned leader and follower roles (Pfister et al., 2014). Participants interacted with key presses which produced tones (see Fig. 1 for trial procedure).

In Experiment 1, the leader performed a key press at a time of their choice, generating a tone. This tone was a “go” signal for the follower to perform a key press, which had to be within a time limit. On each trial, each participant estimated either the Leader Action-Tone Interval (Interval 1) or the Tone Follower-Action Interval (Interval 2). In Experiment 2, the follower’s key press also generated a tone and this new Follower Action-Tone Interval (Interval 3) was also estimated.

Temporal Binding was consistently exhibited by the leader for their own Action-Tone Interval, which was as predicted (Pfister et al., 2014). Interestingly, leaders also showed binding for the Tone-Follower Action Interval, but only when that action produced its own subsequent tone (Pfister et al., 2014). Pfister et al. (2014) propose that as the leader could choose when to initiate the interaction and their action then prompted the follower to act, this induced vicarious agency over the follower and their action (Pfister et al., 2014). In support, leading or following has been demonstrated to activate different parts of the right intraparietal sulcus (Chaminade & Decety, 2002), associated with the intention and action monitoring components within Sense of Agency (Pfister et al., 2014; Sidarus, Vuorre, & Haggard, 2017).

Arguably, the most interesting component of the Pfister et al. (2014) study is the opposing agency effects for the leader and follower roles. The follower never exhibited binding effects for their own or leader action intervals, and no binding was exhibited from either participant for the Follower Action-Tone Interval (3). The follower’s volition was restricted, resulting in ‘violated’ agency, in this paradigm by multiple elements: the overt label of follower, only acting in response to prior actions in each trial sequence, reacting to a prescribed ‘go’ signal, and the time limit in which to perform the action. This is against prediction, as even without intentionality, the causal links between action and tone should still induce some degree of binding effects (Buehner, 2015; Buehner & Humphreys, 2009; Imaizumi & Tanno, 2019; Moore & Obhi, 2012; Suzuki, Lush, Seth, & Roseboom, 2019). It appears that this violation of agency is powerful enough to override these effects. When evaluating this study, there must also be consideration that the effect of both the leader and follower action is not a direct social response; the interaction is mediated by tones. This not only creates more temporal disparity between actions, but may diminish the perception of how *social* the interaction actually is, impeding wider inferences relating to Social Agency.

The findings of Pfister et al. (2014) have been replicated in other paradigms which defined leader and follower roles (Bolt et al., 2016; Reddish et al., 2020). When a leader dictated the speed of a synchronous arm movement with a follower, the leader experienced vicarious agency over the follower’s actions, and conversely, the follower attributed the agency of their actions to the leader (Reddish et al., 2020). What is interesting about Reddish and colleagues’ (2020) result was that the same task with egalitarian roles was found to induce shared agency (see section titled Shared Agency ). Even when engaging in coordinated action, which generally induces shared agency, introducing *asymmetrical* coordination through leader and follower roles influences how shared or independent each actor rates their own actions. Leaders report independent agency, whilst followers report shared agency, negating their own agentic status (Bolt et al., 2016). This demonstrates that even small differences in an actor’s role within the same interaction modulates Social Agency effects.

### Interfered agency

Most of our social interactions are loose interactions, whereby a joint goal is not defined beyond exchanging some information or having a nice time together. Typically, such situations may elicit entrainment or other dyadic alignment, but there is rarely any ambiguity about who caused a specific joint action effect, because very often there isn’t one. Instead, within the dyad the agents act and react to one another and their actions hence become both causes and effects, often within an overarching nonlinear dynamic.

When interacting with an agent who appears independent (i.e. little or no cooperation), our agency appears to diminish. When participants were led to believe that they were interacting with another person, their explicit agency ratings were reduced (Beyer et al., 2017), and it elicited different neural activity from that associated with nonsocial Sense of Agency (Beyer et al., 2018). Social contexts were associated with an increase of activity in brain areas responsible for mentalizing processes, including the precuneus (Beyer et al., 2018). The precuneus is most consistently active in mental imagery tasks, including visuospatial perspective taking (Schurz, Radua, Aichhorn, Richlan, & Perner, 2014). The authors proposed a model for Sense of Agency where the presence of another independent agent, making their own actions, interferes with action selection (i.e. self-action planning processes; Beyer et al., 2017; Beyer et al., 2018). It is this interference, or action disfluency, that is argued to reduce agency (Beyer et al., 2017; Beyer et al., 2018; Haggard, 2017; Metcalfe & Greene, 2007).

A way of improving the agentic status of an actor is to give them an action choice (Pfeiffer et al., 2012). This study investigated the effect of social response latency and presence of joint attention on an explicit measure of Social Agency (Pfeiffer et al., 2012). Participants were seated in a room with a confederate with whom they believed they were interacting behind a partition. They were presented with two everyday objects on either side of a central anthropomorphic avatar whom they believed was controlled by the eye movements of their interaction partner. On each trial, participants fixated on the avatar, then chose which object to saccade to. After a variable interval, the avatar would then either follow gaze to the same object or avert gaze to the other object, establishing joint attention or nonjoint attention, respectively. Participants were then asked to rate the relatedness of the other’s action to their own on a 4-point scale, from *very related* to *very unrelated*. Results demonstrated gaze following was rated as related to the participant’s action, whereas gaze aversion was rated as unrelated; ratings were consistent across latencies. Pfeiffer et al. (2012) explain these results in relation to valence. As initiating joint attention is seen as intrinsically rewarding and positive (Frischen, Bayliss, & Tipper, 2007; Grynszpan et al., 2017), this would explain the increase in relatedness ratings. Conversely, the authors argue that gaze aversion is viewed as negative, which would explain the decrease in the relatedness ratings. However, it is limiting to interpret the results only in terms of valence. Results could suggest that participants were attributing vicarious agency (see the section titled Vicarious and Violated Agency) to actions which followed their own, with gaze aversion being attributed to the other’s agentic status. Both these attributions may explain why relatedness ratings are consistent, even as temporal contiguity decreases: the other is understood to be making a volitional temporal choice, with or without agentic status (Pfeiffer et al., 2012). This extended inference ties in with a follow-up experiment which was conducted where the action of the other was restricted to always follow the gaze of the participant. In this experiment, agency decreased linearly beyond a short latency (Pfeiffer et al., 2012). With no action choice, the agency attributions (vicarious or agentic) diminish as temporal contiguity decreases, in line with nonsocial findings (for review, see Wen, 2019).

Exhibiting a diminished Sense of Agency in social interactions when interacting with an independent agent may also be an underlying factor in wider behavioural phenomenon relating to diffusion of responsibility. Behaviourally, diffusion of responsibility has been demonstrated to reduce an individual’s likelihood of assisting in an emergency (The Bystander Effect; Barley & Latanfi, 1968) and effort invested in group tasks (social loafing; Williams et al., 1993), whilst conversely increasing aggression (Bandura, Underwood, & Fromson, 1975) and risk taking (Teger & Pruitt, 1967). This reduction in the Agency while acting in social contexts, observed in the behavioural phenomena associated with diffusion of responsibility, supports Beyer et al.’ (2017) model of Social Agency. For a concise summary of the types of Social Agency covered in the section titled Types of Social Agency, see Table 1.

**Table 1:** Summary of types of Social Agency, including both an everyday and empirical example

Social Agency type	Everyday example	Study example	Explanation/Details
‘We’ agency	In a room with more than one entrance, two people entering, flicking different light switches simultaneously, and wondering who actually caused the light to turn on	Obhi and Hall (2011a)—Dynamic condition where who acted first changed trial-wise, increasing ambiguity over who cause the tone 200-ms later	• Joint action • Formation of a joint agentic identity between parties • Egalitarian • Blurring of the self/other boundary
Shared agency	Two people repositioning a sofa within a room	Stephenson et al. (2018)—Initiating joint attention with another on an intended object	• Joint action • Formation of a joint agentic identity between parties • Egalitarian • Duality of the joint identity and of self-identity
Vicarious agency	At work, instructing a subordinate staff member to do a task	Pfister et al. (2014)—Leader role in the action/tone interaction sequence	• ‘Leader’ roles with free action to initiate interaction • Misattribution of action of another (follower) to self • Hierarchical contexts, distinct roles
Violated agency	A superior staff member instructing you to execute a task at work	Pfister et al. (2014)—Follower role in the action/tone interaction sequence	• ‘Follower’ roles where there is a restriction of own action in response to an action of another • Restricted volition resulting in not attributing own action to self • Hierarchical context, distinct roles
Interfered agency	‘How are you?’—Asking an open question whit no expected response or more than one plausible predicted answer	Beyer et al. (2017)—Making an action to prevent a negative outcome in the presence of another agent who may or may not also act, compared with the same action alone	• Other without expected action, seen as an independent agent • Process of predicting other action interferes with own-action process • Increased cognitive load • Only abiding by social norms (e.g. prosocial response of gaze following) causes any attribution of other’s action to own agency

## Social Agency continuum

**Fig. 2:**
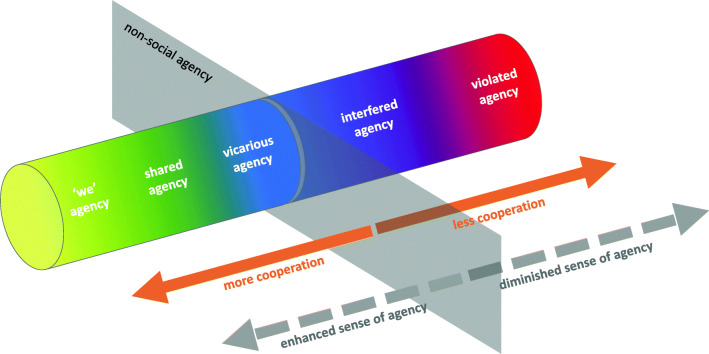
Depiction of Social Agency as a continuum of cooperation with enhanced or diminished Sense of Agency effects in comparison to nonsocial agency. Each type of Social Agency that can be induced in an interaction is shown as a different colour along the continuum (e.g. ‘we’ agency as yellow). The colours deliberately merge to illustrate that these types of Social Agency are not clearly defined categories; the manipulation of one of more interaction elements can shift the kind of agency induced from one to another

The above evaluation of the evidence related to Social Agency allows us to develop a novel picture of the relationship between the different types of Social Agency. We propose Social Agency as a continuum, where various elements of cooperation (e.g. predictability of others’ actions) within a social interaction may enhance or diminish Sense of Agency to varying degrees, resulting in ‘we’, shared, vicarious, violated, or interfered agency. Figure 2 depicts the proposed continuum, comparing where agency types sit in relation to enhanced or diminished effects. Nonsocial (i.e. environmental) Sense of Agency is used as a theoretical neutral position for comparison. The types of Social Agency are depicted as merging colours to emphasize that Social Agency is a complex construct, where conflicting influences or interaction elements mean that there are no clean-cut boundaries between the various types; many interactions can move from one Social Agency type to another by simply manipulating one element. As discussed above, most of the extant literature reviewed scrutinizes Social Agency (joint) and (context)—therefore, this continuum is based mainly on these instances. With little empirical evidence for Social Agency (effect), it is not as clear how this instance fits into the model. Conservatively, we can speculate that prosocial responses from another would enhance agency (Pfeiffer et al., 2012; Stephenson et al., 2018), whilst antisocial responses would diminish agency (Pfeiffer et al., 2012). It is also important to note that this continuum is not a scale and that Social Agency is not one dimensional. As will be discussed later in this section, there are many interaction dimensions critically underresearched in relation to Social Agency, and whilst this continuum is centred around the degree of cooperation in an interaction, as Social Agency grows as a field, it is hoped that more key elements will be incorporated into this model.

Egalitarian social interactions with higher degrees of cooperation are argued to enhance the Social Agency compared with nonsocial agency. Cooperation is of distinct evolutionary and sociocultural benefit, so this comes as no surprise. We speculate that feeling an enhanced Sense of Agency in interactions with mutual goals, responsiveness, and outcomes promotes further engagement in these types of interactions. This aligns with the proposal that humans have a predisposition for cooperation, motivated by “the rewarding nature of the active participation in social interactions” (Pfeiffer et al., 2014, p. 135). As multiple parties can achieve larger goals or goals more quickly than any individual, this helps in developing human interdependence and a speed of cultural growth far beyond any other species. This phenomenon, known as Cumulative Culture Evolution, sees generational gains in cultural complexity more than what any individual could achieve (Mesoudi & Thornton, 2018).

This review argues that the most enhancing type of Joint Agency is ‘we’ agency. Above and beyond the forming of a shared agentic identity (as in shared agency), ‘we’ agency has an additional component of blurring the boundaries between self and other (Pacherie, 2012). This blurring of the self is experienced not only positively but also pleasurably (Wahn, Kingstone, & König, 2018). The sociological theorist Emile Durkheim regarded this blurring as something called ‘collective effervescence’, where groups can have a heightened collective emotional excitement not found in individuals (Gabriel, Naidu, Paravati, Morrison, & Gainey, 2020). As an example, imagine the enjoyment of going to a sold-out concert and ponder whether the same music would sound as good in an empty stadium. This blurring could mean that agency expands vicariously to all persons present. Experimentally this has so far only be demonstrated to extend to one other, but theoretically this could be the same for any number of parties.

As discussed in the section titled ‘We’ Agency, synchrony is a phenomenon which can help induce ‘we’ agency. As humans have social networks larger than would be expected by our brain size, mechanisms such as synchrony facilitate social bonding (Tarr et al., 2016; Tunçgenç & Cohen, 2016), which encourages group cohesion (Launay et al., 2016). Social synchrony has been demonstrated in the synchronization of different body movements (see Richardson & Johnston, 2005; Schmidt, Carello, & Turvey, 1990) and is pervasive to an extent that it can even override the natural (eigen)frequency of a rocking chair when sitting next to someone (Richardson, Marsh, Isenhower, Goodman, & Schmidt, 2007). This ties in with the benefits of cooperative social interactions and why we jointly experience such interactions (Sebanz et al., 2006). It is even possible that mutual synchrony changes an interaction from being perceived as one with independent agents to one with ‘we’ agency, as in Reddish et al. (2020).

Whilst shared agency can also enhance Sense of Agency, the concept of the Self is still intact, and as such, is a modulator for the degree of agency experienced. This is why shared agency is depicted in the continuum as enhancing, but to a lesser extent than ‘we’ agency. Shared agency arises once again through a degree of cooperation, in prosocial actions such as leading gaze to initiate joint attention (Frischen et al., 2007; Pfeiffer et al., 2014) or mutual coordination (Bolt et al., 2016), but in these circumstances, there is no ambiguity over action origin. Initiating joint attention is known to recruit activation of the ventral striatum, a brain area associated with reward-related neurocircuitry (Pfeiffer et al., 2014; Rilling & Sanfey, 2011). This area is exclusively activated in *social* contexts; nonsocial interaction only activates attentional networks (Pfeiffer et al., 2014). This demonstrates the uniquely rewarding nature of social interactions (Pfeiffer et al., 2014), which enhance Social Agency (Pfeiffer et al., 2012).

The next two types of agency to discuss are somewhat linked. Vicarious agency is enhanced agency through attributing other’s actions to the Self, whereas, conversely, violated agency is where no or little agency is experienced for self-actions. These regularly manifest in the same paradigm, by the introduction of hierarchical roles within an interaction. All of the studies reviewed use leader and follower roles to induce vicarious and violated agency, respectively (Bolt et al., 2016; Pfister et al., 2014; Reddish et al., 2020). Whilst there can be cooperative elements to an interaction, the change in power dynamic is a more salient element in relation to how people experience agency. With distinct roles, the Self is very much intact, and therefore vicarious agency is depicted as having enhancing effects similar to shared agency, perhaps slightly less so. Violated agency is depicted as having the most diminished Social Agency, since an important component of agency, volition, is compromised. It is interesting that both subordinate and dominant actors view the subordinate’s agency as diminished (Pfister et al., 2014). This could also explain why robotic interaction partners may not be attributed with an agentic status (see section titled Social Agency With Artificial Agents); actions are perceived to be preprogrammed and not generated through free will.

Interactions with little or no cooperation also diminish Social Agency, as having to predict actions in these circumstances is much harder, interfering with self-agentic processes. Without any consensus between parties as in cooperative interaction, conspecific effects (i.e. the responding actions of another) are far less predictable than environmental effects, and hence engender diminished Social Agency. As the empirical evidence demonstrates, there may once again be vicarious agency from an initiator in an interaction over the response of another, but that this is only exhibited when the response is a *chosen* prosocial response (as in Pfeiffer et al., 2012). As we are a socially driven species, when no other predictive cues can be relied upon, the expectation of another performing a prosocial response prevails (Grynszpan et al., 2017), and a positively valenced self-serving bias attributes this to the initiator (Wang et al., 2017). Conversely, chosen antisocial responses are attributed to the agentic status of the other (Pfeiffer et al., 2012). This is interesting because it is something which has been demonstrated to be capable of changing dynamically (Pfeiffer et al., 2012).

From the limited empirical evidence and continuity with other social psychology and sociological theories, this review argues that viewing Social Agency as a continuum mediated by degree of cooperation is the model which best fits what we currently know. That being said, it is important to acknowledge the limitations of this theory; there are still a host of unexplored interaction dimensions within Social Agency (see Box 1 for an overview of social interaction dimensions). For example, how Social Agency is influenced in larger-scale group interaction is completely unknown; current empirical investigation focusses exclusively on individual (dyadic) interactions. Even within this individual parameter, there is a scarcity of studies with clearly defined roles that would replicate a hierarchical interaction structure. Also, within cooperative contexts the association between partners has so far always been of a novel, transient nature, limited to that experiment. Pacherie (2012, 2014) identifies the longevity of association between partners to be a key dimension of cooperative interaction. Hence, establishing paradigms with repeated sessions or using participants with preexisting rapport would allow testing the influence of longer-term associations. Furthermore, it remains to be seen to what degree Social Agency is determined by the relative pivotality of each agent in a cooperation. Pivotality, which refers to the degree to which an individual can impact the outcome, can be influenced by several factors, like roles, symmetry, or synchronicity of actions. In a recent paper, Le Bars et al. (2020) show that individual versus collective Judgements of Control (JoC) are differentially sensitive to manipulations of agents’ pivotality.

**Table:** 

**Box 1 Dimensions of social interaction**		
**Structural dimensions**		
Physical	< - - - >	Virtual
Cooperative	< - - - >	Independent
Individual	< - - - >	Group
Small-scale	< - - - >	Large-scale
Generalized	< - - - >	Specialized
Egalitarian	< - - - >	Hierarchical
Equal pivotality	< - - - >	Unequal pivotality
Transient	< - - - >	Stable
Normative	< - - - >	Novel
Simple	< - - - >	Complex
**Motivational dimensions**		
Self	< - - - >	Group
Cooperative	< - - - >	Competitive
Goal	< - - - >	Enjoyment
Adapted and expanded from Pacherie’s (2012, 2014) work on joint actions, dimensions of social interaction are defined and categorized into structural or motivational aspects. Little is still known about how each of these dimensions influence Social Agency and if the influences of different dimensions are congruent or conflicting with each other. With social interactions being capable of involving dimensions on different levels of functionality, opposing ends of the same dimension may also be within one interaction. Take, for example, a chess game: There has to be a certain level of *cooperation,* where players take turns to make a move and adhere to the rules, but there is also a *competitive* element, as each player wants to win. What defines the interaction is ultimately the level which has the strongest motivational element (see Cho et al., 2020), so in the instance of a chess game, the competitive influence will be stronger than the cooperative.		

There is also a scarcity of studies scrutinizing competitive social contexts (see Cho, Escoffier, Mao, Green, & Davis, 2020). With competition being another aspect intrinsic to human society, it is of utmost importance that we learn more about how this affects Social Agency. Competition brings with it conflicting agency influences which are identified in this review: The positive self-bias could enhance Sense of Agency in victory and diminish in defeat, but the nature of competitive contexts could enhance self–other distinction, increasing interference and diminishing agency even more. Group contexts would complicate things further by introducing levels of both cooperation and competition to the same interaction, where individuals work together cooperatively to then compete as one identity against another. As joint cooperative–competitive contexts are prevalent in all aspects of life, from recreational sports to employment, this would offer a highly relevant aspect to conduct research on.

## Factors influencing Sense of Agency

Focus now moves from the currently unexplored dimensions of Social Agency to questions which arise from the existing literature. With there being widespread variation in measures of Sense of Agency, and recognized conditions which need to be satisfied for Sense of Agency to arise (e.g. temporal contiguity, predictability), it is important to evaluate experimental factors which may also influence Sense of Agency. This might be especially pertinent in Social Agency paradigms as experimental manipulation will allow systematic evaluation of the contributions of top-down (i.e. perceptual) or bottom-up (i.e. sensory) processes. The factors detailed below are those which differ in influence or importance for social, compared with nonsocial, Sense of Agency, highlighting again why Social Agency in itself is a distinct construct. The factors are synthesized into Table 2 to aid comparison, split into columns for Nonsocial Agency and Enhanced and Diminished Social Agency.

**Table 2:** Manipulated experimental factor and their implicit and explicit effects for nonsocial Agency, enhanced Social Agency, and diminished Social Agency

Manipulated factors	Nonsocial Agency		Social Agency			
			Enhanced Social Agency: ‘we’, shared, or vicarious		Diminished Social Agency: violated or interfered	
	Implicit	Explicit	Implicit	Explicit	Implicit	Explicit
Temporal contiguity	•Sensory attenuation decreases >200 ms (Blakemore et al., 1999) •TB results diverge depending on methodology (see section in review)	•SoA ratings linearity decreases as delay increases (Wen et al., 2015)	**•UNKNOWN—**Manipulated in Pfeiffer et al. (2012) 100–600 ms and Stephenson et al. (2018) 400–2,300 ms, but effects on TB specific to this not reported	**•UNKNOWN—**Manipulated in Stephenson et al. (2018) 400–2,300 ms, but effects on SoA ratings specific to this not reported	**•UNKNOWN**	•Temporal contiguity (0–4,000 ms) no longer a condition of SoA when ‘other’ has an action choice (Pfeiffer et al., 2012)
Action choices	•TB linearly increases as action options increase (Barlas & Obhi, 2013)	**•UNKNOWN**	**•UNKNOWN—**Predicted to follow nonsocial implicit trend for both explicit and implicit measures, but would be less relevant in cooperative contexts, where there are mutual goals. It may even be that giving an action choice makes consensus of those goals more difficult, hindering SoA compared with contexts		**•UNKNOWN—**Predicted to follow nonsocial implicit trend for both explicit and implicit measures, but be of importance in less cooperative contexts in relation to establishing agentic status to ‘other’. This may result in a stronger linear increase as action options increase	
Outcome control	•Motivational significance of outcome modulates TB effects (Beck et al., 2017) •Unexpected outcome may induce ‘hyper’ TB (Majchrowicz & Wierzchoń, 2018)	**•UNKNOWN**	•TB effects for both the initiator of the outcome and a responder who knew they did not cause the outcome in a joint-action task (i.e. shared agency effect; Obhi & Hall, 2011)	•Explicit SoA experienced only by the initiator of the outcome in a joint action, no the responder, who did not contribute to the outcome (Obhi & Hall, 2011)	**•UNKNOWN**	•When ‘other’ given an action choice (outcome control removed for participant), ratings of relatedness of this action to own diverge: congruent actions are rated highly related, incongruent highly unrelated (Pfeiffer et al., 2012)
Initiating joint attention			•Increased TB for gaze leading, compared with control passive face (Stephenson et al., 2018)	•Agency ratings increased for gaze leading, compared with control passive face (Stephenson et al., 2018)	**•UNKNOWN**	•When the ‘other’ has an action choice, initiating JA increases ratings of relatedness of this action to own. Conversely, when the other’s only action is to gaze follow, ratings of relatedness to this action decrease linearly as latency of gaze following increases (Pfeiffer et al., 2012)

There are several other factors which are reported to influence Sense of Agency more generally and should also be considered; these include physical effort (Howard, Edwards, & Bayliss, 2016), cognitive load (Hon, Poh, & Soon, 2013; Howard et al., 2016), coercion (Caspar, Christensen, Cleeremans, & Haggard, 2016), and prior bias or priming (Cravo, Haddad, Claessens, & Baldo, 2013; Sidarus, Chambon, & Haggard, 2013; Sidarus et al., 2017). However, this review will not incorporate these factors due to lack of evidence in relation to Social Agency effects.

## Temporal contiguity

Temporal contiguity (or temporal predictability; Ruess, Thomaschke, & Kiesel, 2017) is of special interest to Social Agency, as social outcomes are reasoned to contain more inherent variance than nonsocial ones, which are relatively immediate (Pfeiffer et al., 2014; Stephenson et al., 2018). Most studies investigating Sense of Agency manipulate temporal contiguity by varying the onset of the effect after action (Cravo, Claessens, & Baldo, 2011; Haggard, Clark, & Kalogeras, 2002; Pfeiffer et al., 2012; Pfister et al., 2014; Stephenson et al., 2018). Some studies use this to evaluate if there is a point of optimal contiguity (MacKenzie, 2013), or a nonlinear time course for Sense of Agency effects (Pfeiffer et al., 2012; Ruess, Thomaschke, & Kiesel, 2018b). Others simply collapse different delay conditions during analysis, using them as experimental jitter to optimize engagement (Stephenson et al., 2018).

One study (Pfeiffer et al. 2012) demonstrated that when the ‘other’ in a social interaction was given their own action choice, temporal contiguity was no longer a condition to experience Sense of Agency. This represents an important difference for Social, compared with nonsocial agency.

This finding was robust for gaze latencies up to 4 seconds, a considerable delay for a gaze-related action. Whilst this suggests the irrelevance of temporal contiguity in social interaction, this finding needs further investigation and replication; one study cannot provide conclusive evidence. This finding is also contrary to the theory that reactivity from a partner, determined by temporal contingency and congruency, strongly influences self-Social Agency (Brandi et al., 2019; Bratman, 1992). How temporal contiguity affects Sense of Agency in cooperative social contexts is still unknown. When cooperating with another, it may be that there is indeed some degree of temporal expectancy for the responding action in line with Brandi et al. (2019), relating to the joint goal. This would also tie in with one of Bratman’s (1992) defined theoretical features of joint actions: mutual responsiveness. The shared goals from joint action are seen to induce circumstances where each party has a reciprocal expectation and duty of responsiveness, relying on cues from each other to achieve this (Bratman, 1992; Pacherie, 2012).

Pfeiffer et al. (2012) only used a somewhat indirect explicit measure of Social Agency. Hence, there are no current predictions of how temporal contiguity may affect implicit measures. One study did manipulate temporal contiguity of action and effect and measured Temporal Binding (Stephenson et al., 2018), but this temporal variation was discarded in analysis by computing the deviance of the reported interval from the actual interval (in terms of percentages) and averaging these across conditions.

A recent review (Wen, 2019) assessed the impact of outcome delay on different Sense of Agency measures in nonsocial settings. It found that whilst explicit judgements decreased linearly as delay increased (Wen, Yamashita, & Asama, 2015) and Sensory Attenuation effects faded when delay exceeded 200 ms (Blakemore, Frith, & Wolpert, 1999) as would be expected, Temporal Binding results diverged. Some studies report the strongest binding at very short delays (150 and 250 ms, respectively; Haggard et al., 2002; Ruess, Thomaschke, & Kiesel, 2018a), whereas other studies report stronger binding as intervals increase (Buehner & Humphreys, 2009; Dewey & Knoblich, 2014; Ruess et al., 2017; Wen et al., 2015). This makes it difficult to predict how temporal contiguity in different social contexts may affect Temporal Binding, given that even results for nonsocial agency do not converge. The review (Wen, 2019) does concede that the binding divergence found may be due to methodological differences, rather than direct influence (Dewey & Knoblich, 2014; Wen, 2019). There may be differences in when action effects are expected, depending on modality of cue and/or outcome. As auditory processing is faster than visual processing (Formby, Morgan, Forrest, & Raney, 1992; Shimojo et al., 2001; Welch et al., 1980), it could be surmised that the expectation of an auditory effect would be faster, leading to the strongest Sense of Agency at shorter intervals. There may also be an effect of action (or task) type. Where tasks require multisensory processing (thus increasing cognitive load), there could be suppressed Temporal Binding for the shortest delays (Hon et al., 2013; Howard et al., 2016). This again highlights the importance of clear design principles and field-wise consensus on methodology to produce comparable measures.

## Action choice and outcome control

Whilst action choice and outcome control concern different processing stages—action selection and action execution, respectively—here, we group them together, as they are both centred round the same concept: control. Sense of Agency arises only when actions are performed voluntarily (Haggard et al., 2002). As action choice is seen as a crucial aspect of agentic experience and enhances control (Wenke, Fleming, & Haggard, 2010), it would also seem reasonable that choosing action from multiple options would enhance Sense of Agency, aligning with wider societal concepts interlinking freedom, choice, and agency (Chen, 2013; Krause, 2012). Action choices increasing Sense of Agency was confirmed by a Libet (2002) clock Temporal Binding paradigm, where it was demonstrated that binding linearly increased as action options increased (i.e. increased choice of which key on keyboard to press in response; Barlas & Obhi, 2013), even though the outcome was consistent. This study is notable because of the stringent design: Action choice was specifically isolated while temporal contiguity and outcome were controlled throughout. As no explicit measure was employed, replicating this design with such a measure would be a pertinent avenue for further research, allowing comparison of the influence of action choice on both implicit and explicit agency. It can be predicted that action choice would increase agency even in social contexts and may even be enhanced further in certain contexts. Within Social Agency, it may be more important to choose an action in interdependent contexts to establish your agentic status to the other. On the other hand, as cooperative contexts induce ‘we’ agency, action choice may be less relevant to agency than outcome choice, which would be congruent or incongruent to the shared aim established by cooperation. It may even be that action choice for the interaction initiator hinders Social Agency as this could increase ambiguity of the mutual goal.

In nonsocial interactions, action choice is employed experimentally as a way of *controlling* outcome, which makes the two elements inextricably linked. In a Temporal Binding paradigm with painful and nonpainful somatosensory outcomes, it was shown that controlling the intensity of outcome through probabilistic discriminative action selection increased binding (both action and outcome shift), and more so with painful outcomes (Beck et al., 2017). The fact that binding was greater for painful outcomes also suggests binding was modulated by the motivational significance of the outcome (Beck et al., 2017). These findings link concepts of volition and evaluation of valanced outcomes (motivation) to agency. Conversely, in a recent Temporal Binding paradigm where a single, controlled action produced a controlled outcome in the majority of cases (83%), unexpected outcomes *increased* binding effects (Majchrowicz & Wierzchoń, 2018). Whilst this finding was contrary to prediction, explicit agency judgements greatly diminished for unexpected outcomes, in line with prediction. Authors speculate that the high saliency of unexpected outcomes, due to scarcity of occurrence, could have induced “hyper” Temporal Binding (Majchrowicz & Wierzchoń, 2018, p. 320). This hyper Temporal Binding was argued to manifest through temporal prediction errors, as when action–effect delay was consistent between controlled and unexpected outcomes and only qualitative properties differed (i.e. frequency and waveform), this effect disappeared (Majchrowicz & Wierzchoń, 2018). However, the authors concede that hyper Temporal Binding effects reduced over experimental blocks as participants habituated to the unexpected outcomes. Again, finding divergence between studies is unsurprising when methodologies vary considerably.

In relation to Social Agency, whilst the experimental aspect of outcome choice *can* be separated from action choice, it also becomes more complex, as it is the responding action of an independent agent. With this in mind, Pfeiffer et al. (2012) discovered that giving an action choice (i.e. experimental outcome choice) to the ‘other’ in a social interaction increased their status as an independent agent. This may inhibit self-agency by disrupting the causal link between the initiating action and the responding action of the ‘other’; the responding action has its own agency. Social outcomes also come with their own intrinsic motivations, which, as discussed above, can modulate Temporal Binding. Prosocial outcomes are inherently desirable, inducing reward-like neural activation (Pfeiffer et al., 2014; Rilling & Sanfey, 2011). Therefore, social outcomes seen as prosocial, such as action following, engaging in joint attention, or gaze following, which are *chosen* by the ‘other’ may enhance binding effects.

## Modality of outcome

Sense of Agency paradigms employ visual, auditory, or somatic stimuli for action outcomes. The effect of outcome modality on Sense of Agency was recently investigated in a meta-analysis of Temporal Binding paradigms (Tanaka et al., 2019). Findings suggested that Temporal Binding effects are more robust for auditory compared with visual or somatic stimuli (Tanaka et al., 2019). This ties in with the inference that somatic effects are less distinct from action production and therefore do not reliably reflect causality (Engbert et al., 2007). The differences in temporal resolution of processing between modalities may also contribute to this finding, with auditory processing known to be faster than visual (Formby et al., 1992; Shimojo et al., 2001; Welch et al., 1980). These findings suggest that relying on auditory stimuli for Temporal Binding measures would be prudent. Differences in modality are also relevant for a deeper examination of action and outcome shifts. Outcome shifts alone are reported for visual stimuli (Ruess, Thomaschke, Haering, Wenke, & Kiesel, 2018), whilst action shifts alone are reported for somatic stimuli (Tanaka et al., 2019). Effect sizes are also larger for outcome shifts in general, across modalities (Tanaka et al., 2019). Taking all of these findings into account, it would be advisable to rely on auditory outcomes for Temporal Binding paradigms and to use time-point judgements for measurement (where possible; see section titled Difficulties in Measuring Social Agency). However, it must be conceded that little is known about outcome modalities for different measures of Sense of Agency, so this reliance cannot be prescriptive without further investigation.

With actions such as eye movements having pervasive social importance, modality of outcomes for Social Agency paradigms will have additional considerations. It may be that the social importance (i.e. social weight) of the outcome modality, may modulate binding effects. Responding to a leading eye movement in kind and initiating joint attention is a strong prosocial signal (Frischen et al., 2007; Pfeiffer et al., 2014; Rilling & Sanfey, 2011). This would be more so than a congruent auditory or somatic action outcome as we are attuned to respond to gaze social cues from infancy (Friesen & Kingstone, 1998). It may be that visual outcomes elicit enhanced binding effects, specifically in social contexts where the action outcome is a congruent eye movement of the ‘other’ to a leading eye movement of the interaction initiator. Accordingly, some of the most prominent Social Agency studies discussed in this review (Pfeiffer et al., 2012; Stephenson et al., 2018) have indeed been conducted using eye-movement interactions.

## Wider Considerations

### Social Agency with artificial agents

With the current state of research on Social Agency still very much in the exploratory stage, and there being many methodological considerations to be taken into account, it is advantageous to broaden horizons when considering future research design. A crucial aspect of social psychology research in general is the ecological validity of paradigms (for example, Albert & de Ruiter, 2018; Hermans et al., 2019; Kingstone, Laidlaw, Nasiopoulos, & Risko, 2017; Reader & Holmes, 2016; Risko, Richardson, & Kingstone, 2016; Schlichting et al., 2018); effort must be made to successfully induce a social context. As most social research is screen-mediated to give more experimental control, virtual characters or preprogrammed human stimuli (e.g. videos, photos) are used to interact with participants (Caruana, de Lissa, & McArthur, 2017a; Gobel, Kim, & Richardson, 2015). Importantly, virtual characters may be believed to be *avatars* with human agency or computer-controlled *agents* (Caruana, de Lissa, et al., 2017a). This distinction is vital when wishing to examine social phenomena.

Investigation into whether anthropomorphic representation (social *appearance*) or human agency (social *relevance*) is required to produce reflexive orienting of visual attention revealed that it is social relevance that drives it (Gobel et al., 2018; Wiese, Wykowska, Zwickel, & Müller, 2012). Performance in a visual cue task was facilitated when participants believed a dot on screen was the representation of another participant’s gaze location, compared with a computer-generated location (Gobel et al., 2018). This finding is made even more relevant by the finding that brain activity elicited by the same avatar stimuli can differ depending on agency beliefs (Caruana, de Lissa, et al., 2017a). Using electroencephalography (EEG), larger event-related potentials (ERPs) in the left occipitotemporal region and exclusive activation within the centroparietal region were observed when reacting to human-controlled, compared with computer-controlled, virtual characters. This relates to Social Agency research in that to truly induce a social interaction, participants should *believe* that they are interacting with another even if this is deceptive (as in Beyer et al., 2017; Beyer et al., 2018; Pfeiffer et al., 2012), rather than paradigms merely presenting stimuli with social appearance (as in Stephenson et al., 2018), which can undermine the validity of social inferences.

The difference in Sense of Agency between human–human and human–computer interaction has been overtly measured in joint-action paradigms (Grynszpan et al., 2019; Obhi & Hall, 2011b; Sahaï, Desantis, Grynszpan, Pacherie, & Berberian, 2019). In a task where outcome attribution was ambiguous between partners, it was demonstrated that interacting with a computer partner failed to induce Temporal Binding effects or explicit agency attributions like those found when interacting with a conspecific; ‘we’ agency can only be induced by human–human interaction (Obhi & Hall, 2011b). Even in conditions where ambiguity is removed and participants *know* the outcome was caused by their action, interacting with a computer in a joint task inhibits binding effects (Obhi & Hall, 2011b; Sahaï et al., 2019). This suggests Joint Agency is human centric (Limerick, Coyle, & Moore, 2014; Sahaï et al., 2019). Hence, establishing the perception of human agency would be essential for screen mediated investigation of Social Agency.

Crucially, the enhancement in Sense of Agency we experience from ‘we’ and shared agency appears to be something we only experience when interacting with other humans. When interacting with nonhuman agents (or robots), it has been found that implicit Social Agency enhancements disappear (Grynszpan et al., 2019; Obhi & Hall, 2011b; Sahaï et al., 2019). Participants also judge their own contribution to joint action as greater when interacting with a robot (Grynszpan et al., 2019). Taken jointly, these findings may seem to suggest that we do not recognize the agentic status of an artificial other, but it is not that simple. Importantly, participants in Grynszpan et al. (2019) were never aware that they were interacting with a robot, always being told they had an unseen human interaction partner. Therefore, the only change between human and robot conditions were subtle differences in haptic feedback from the ‘other’ (Grynszpan et al., 2019). Conversely, diminishing Social Agency effects from interacting with robots mirror those found in human interaction (e.g. interfered agency; Ciardo, Beyer, De Tommaso, & Wykowska, 2020; Ciardo, De Tommaso, Beyer & Wykowska, 2018). Taken together with Grynszpan et al.’ (2019) findings, it suggests Sense of Agency effects in human–computer interaction are not always driven by the agentic status of the other (i.e. top-down processes), but also bottom-up sensory feedback or increased cognitive workload (Ciardo et al., 2020; Ciardo et al., 2018).

### Social Agency and eye gaze

Many social cues and responses are linked to the eyes and eye movements are powerful social cues appropriate to use in the methodology within Social Agency paradigms. This is because our eyes are arguably the foremost sensory organs that both interpret and transmit information (Risko et al., 2016), which makes them attuned to playing a key role in social interactions. A key example of social cues employed in social interactions are leading eye movements (i.e. ‘gaze cues’; Friesen & Kingstone, 1998), which direct an observer to a desired object, location, or person. Gaze cues hold mutually beneficial social consequences for the performer and observer alike: initiating joint attention (Frischen et al., 2007), and facilitating mental state attributions (Gobel et al., 2015, 2018; Nummenmaa & Calder, 2009; Pfeiffer, Vogeley, & Schilbach, 2013).

When conducting Social Agency eye-movement paradigms (as in Pfeiffer et al., 2012; Stephenson et al., 2018), there should also be consideration of the aforementioned fact that in social contexts, gaze has dual function: perceiving and signalling (Gobel et al., 2015). Critically, whether an individual’s eye movements are or are not signalling to another can change gaze behaviour. Participants are less likely to look at conspecifics (Kuhn, Teszka, Tenaw, & Kingstone, 2016; Laidlaw, Foulsham, Kuhn, & Kingstone, 2011), or follow gaze cues (Gallup, Chong, & Couzin, 2012) in real-world interactions where the other can observe them, compared with the same interactions in a screen-mediated laboratory setting, where they know their eye movements cannot be observed. This difference is pertinent to Social Agency as it has been demonstrated that direct eye contact from a face stimulus on screen can enhance Temporal Binding effects (Ulloa, Vastano, George, & Brass, 2019). Eye contact is seen to increase self-referential processing, eliciting increased self-awareness (Baltazar et al., 2014) and therefore implicit agency effects (Conty, George, & Hietanen, 2016). As this manipulation was without human agency behind the face stimulus or signalling of the participants gaze (Ulloa et al., 2019), it would be of interest for future investigations to introduce these elements, which may improve ecological validity and enhance agency effects even further.

## Summary and conclusion

As the field of Social Agency is still in its infancy, this review aimed to both synthesize current knowledge into a theoretical concept and make recommendations for future research. First, we argued that Social Agency must be regarded as distinct from nonsocial Sense of Agency, and we identified three dimensions of Social Agency in terms of social effect (effect), joint action (joint), and simply social presence (context). Second, we argued that the current view of Social Agency is oversimplified, which can account for the disparity of findings in the field. Using an evidence-based approach, we built an argument that Social Agency must be considered a continuum, centred on the degree of cooperation within an interaction (visualized in Figure 2). This conceptualization leads to categorizing Social Agency into ‘we’, shared, vicarious, violated, and interfered agency. This captures the range of evidence available in the current literature, mainly centred on Social Agency (joint) and (context).

By adopting this perspective, Social Agency research offers exciting opportunity for development. A large proportion of the existing research is centred on cooperative interaction between two individuals with transient or novel association. This type of interaction is widely evidenced to enhance Social Agency effects, modulated by the degree of cooperation, but this addresses only part of the continuum. Social Agency effects in more general interactions where the action of the responding agent is less predictable are critically underresearched. With the work of Beyer et al. (2017; Beyer et al., 2018) demonstrating that unpredictable interactions interfere with ones’ Social Agency, it is important that this work is expanded on. Individual and group competitive interactions are also contexts which are ubiquitous in everyday life, but as yet how these affect Social Agency is unknown. Table 2 highlights that many experimental factors which are demonstrated to affect Sense of Agency in general are yet to be explored in relation to Social Agency. These, along with ecological validity considerations should also motivate future paradigms. Overall, this review argues that whilst we still know very little about Social Agency, with an adjustment of perspective, evidence from current literature not only begins to form a more cohesive picture, but leads the field to which empirical questions should be addressed next and identifies Social Agency (effect) as critically under-researched.
